# Information and Communication Technology-based Assessment for Children with Developmental Needs: Kids Brain Balancer

**DOI:** 10.31662/jmaj.2024-0013

**Published:** 2024-08-09

**Authors:** Tomoko Sugiyama, Keiji Hashimoto, Nobuyuki Kawate

**Affiliations:** 1 Department of Rehabilitation Medicine, Showa University School of Medicine, Yokohama, Japan

**Keywords:** Neurovisuomotor development, Intellectual assessment, Information and Communications Technology, Digital application, Special education

## Abstract

**Introduction:**

This study examined the test-retest reliability of the Kids Brain Balancer, a tablet-based cognitive assessment app, among children in the special education system and gathered preliminary validity evidence by evaluating score agreement with the Wechsler Intelligence Scale for Children, Fourth Edition (WISC-IV).

**Methods:**

A total of 36 children undergoing special education (aged 7-11 years) completed the Balancer tasks more than three times for over 1 month. Intraclass correlation coefficients (ICCs) facilitated the analysis of score reliability across sessions. Score agreement with Wechsler indices were evaluated for each task.

**Results:**

Of the nine tasks, six demonstrated moderate-to-good reliability for raw or age-adjusted scores. The Full-Scale Intelligence Quotient (FSIQ), composite scores on the WISC-IV, and Balancer index scores on several tasks exhibited moderate-to-strong correlations over three repeated test administrations. Agreement with the FSIQ varied; however, most visuospatial/executive tasks initially correlated better, whereas verbal/working memory tasks converged by the third session. Those with lower baseline scores exhibited improvement in agreement over repeat testing.

**Conclusions:**

This study provides initial evidence supporting the validity and test-retest reliability of the Kids Brain Balancer in evaluating intellectual/cognitive functioning among children undergoing special education. Enhancement and wider testing could establish this convenient tool to support evaluation of diverse developmental needs.

## Introduction

The prevalence of neurodevelopmental disorders (NDDs) in children has increased due to improved identification and expanded definitions within the Diagnostic and Statistical Manual of Mental Disorders, Fifth Edition (DSM-5) ^(1), (2)^. Published in 2013, the DSM-5 consolidated traditional developmental disability categories into broader NDD diagnoses, such as intellectual disability (ID), autism spectrum disorder, attention deficit/hyperactivity disorder, and learning disorders, while conceptualizing cognitive impairment independently of the ID criteria. This shift highlights the complexity within NDD diagnoses, challenging the provision of optimal interventions solely based on rough intellectual assessments.

In the Japanese education system, children with suspected developmental delays are placed in special needs or after-school special education classes. However, not all receive formal NDD diagnoses, leading to diverse intellectual levels. In addition, the system in Japan further complicates matters by providing special needs education across a wide range of settings, from public schools to private after-school programs, creating diverse pathways. It is essential to formulate individual developmental support plans in special education systems and understand the student’s intellectual level and cognitive ability. Given this situation, the need for appropriate intellectual assessments is increasing to tailor effective interventions in educational settings.

Although various intelligence tests exist, their practical application remains challenging. For instance, the widely used Wechsler Intelligence Scale for Children, Fourth Edition (WISC-IV) ^(3)^, has a long administration time (approximately 1-1.5 h) and must be administered by a trained psychologist or speech-language pathologist ^(4)^. Qualified examiners may not be readily available at all facilities. Ideally, all children receiving special education would undergo standardized intellectual assessment, such as the WISC-IV. However, this is often impractical because of the increasing demand for special needs services. Moreover, existing intelligence tests do not allow for frequent reassessments over short periods to quantify rehabilitation and learning effects. Considering the situation, a simple yet valid cognitive assessment tool could prove valuable for supporting the development of children with varying intellectual abilities and backgrounds. Furthermore, an accessible, cost effective, and reliable test would help meet the demand for practical intelligence assessment methods.

Addressing this need, information and communications technology (ICT) has greatly impacted healthcare efficiency. The “Kids Brain Balancer” (Balancer), a digital application developed by LEDEX Corp., uses ICT to evaluate children’s cognitive function ^(5)^. A key advantage of ICT-based assessment tools like the Balancer is their ability to automatically log detailed performance data during administration, such as reaction times, task engagement duration, and score fluctuations, across individual tasks. This granular tracking enables the evaluation and quantification of rehabilitation progress and learning effects over time. This progress cannot be readily captured by traditional, single-time-point intelligence tests. However, several research questions regarding the practical application of the Balancer still need to be addressed. While the initial study reported moderate-to-strong correlations between the Balancer and WISC scores, with certain Balancer tasks exhibiting high sensitivity and specificity ^(6)^, crucial questions regarding the Balancer’s test-retest reliability as an intelligence assessment tool remain. Test-retest reliability measures the consistency and stability of the scores of an assessment tool over time, indicating whether it produces consistent and reproducible results when administered to the same individuals under similar conditions at different points. A high test-retest reliability coefficient demonstrates the ability of the tool to provide accurate measurements and allows the researcher to interpret scores relative to norms. Repetition of the same test over a brief period may lead to a learning effect, which could impact the test-retest reliability. However, this was not investigated in the previous study. This study aimed to examine the Balancer’s reliability, establishing a foundational understanding of its potential as an intellectual test for children.

## Materials and Methods

### Participants and testing protocol

This study recruited children receiving special education services from various settings (special education school, special education classes, after-school special education service) (n = 36, aged 7-11 years) (mean, 7 years and 11 months; standard deviation, 1 year and 11 months; 19.4% female). Participants were included regardless of their specific abilities in reading and understanding numbers or their motor impairment to assess children across a broad range of intellectual capacities. However, those with significant cognitive impairments, severe receptive communication deficits, or behavioral challenges that would interfere with following verbal instructions were excluded from the study. To evaluate the test-retest reliability, the children completed the same task three or more times within 1 month of the initial testing. Tasks with insufficient repeat testing (Cubic Fit, Initial Letter, Who Am I?, and Same-Game) were excluded from the analysis.

Ethical approval was obtained from the Human Ethics Committee of the relevant university (approval number: 2023-174-B), and written consent was obtained from each participant and their guardians.

### Kids Brain Balancer

The Balancer, a digital application for iPads (Apple, CA, USA) or other tablets, comprises 13 tasks across 4 cognitive domains were as follows:

・Visuospatial skills (Count the Blocks, Cubic Fit, Same Shape, and Drag Race)

・Language abilities (Initial Letter, Which Picture?, Who am I?, and Matching Words)

・Executive functioning, including attention and inhibition (Catch the Mole, Same-Game, Speed Touch, and Follow the Order)

・Working memory (Flashing Lights)

**Figure 1.**
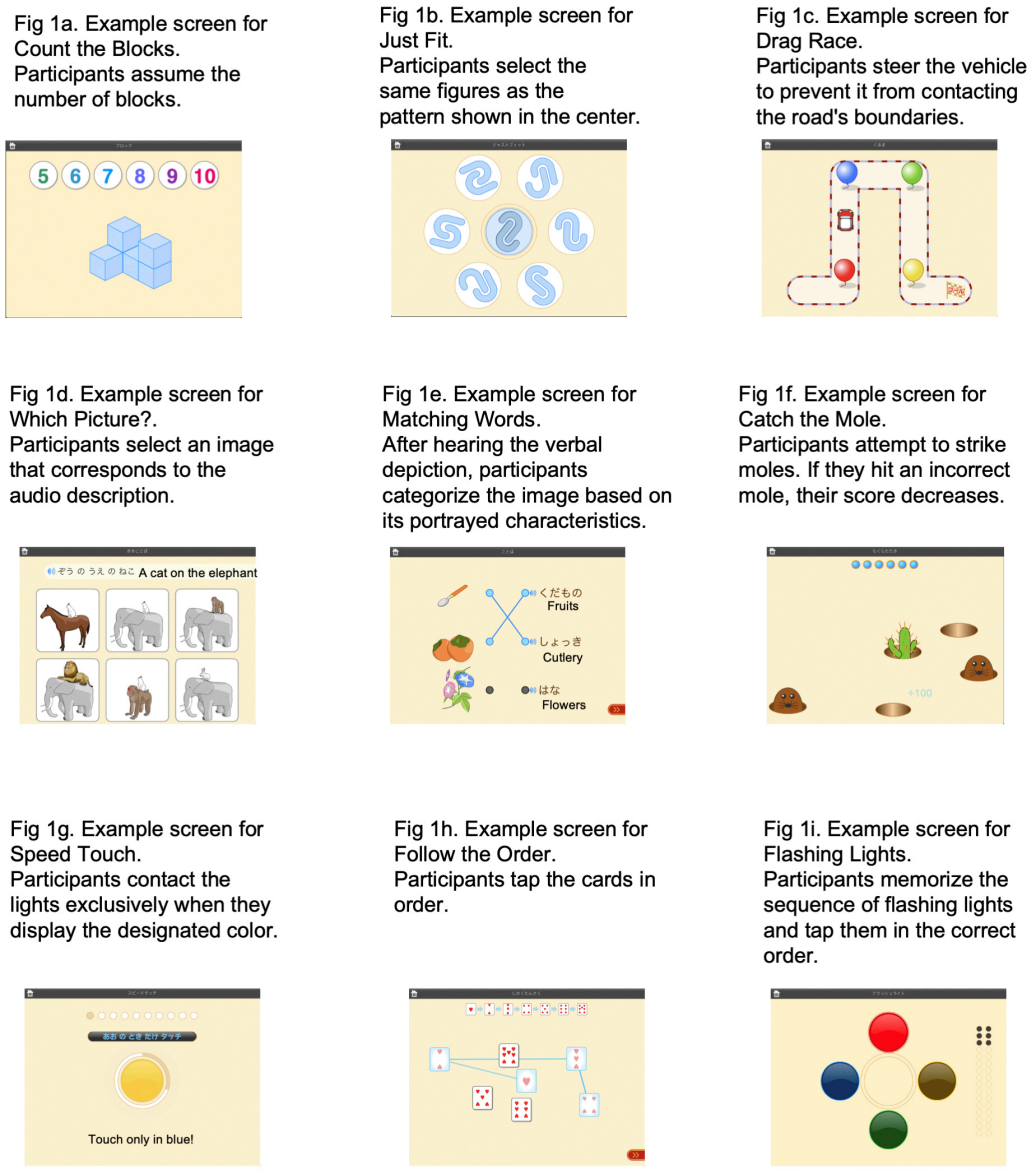
Examples of the screen for representative tasks on the Kids Brain Balancer.

In addition, Catch the Mole and Drag Race tasks involve motor skills. The raw scores for each task were calculated based on the completion time and accuracy, adjusted for age and converted into index scores. Example screens from representative tasks are shown in Figure 1.

### Wechsler Intelligence Scale for Children (WISC)

The WISC-IV is an intelligence assessment for children aged 5-16 years. It comprises 10 core and 5 supplementary subtests. The participants completed both the Balancer and WISC-IV in the same setting. The WISC-IV yields a Full-Scale Intelligence Quotient (FSIQ) along with four composite scores: the Verbal Comprehension Index, Perceptual Reasoning Index (PRI), Working Memory Index (WMI), and Processing Speed Index.

### Statistical analysis

The test-retest reliability, assessed over three administrations per task using the intraclass correlation coefficient (ICC) and 95% confidence interval, demonstrated good-to-excellent reliability. The ICC was estimated using a two-way mixed-effects model with absolute agreement applied to both the raw and index scores. ICC values from 0.90 to 1.00 indicate excellent reliability; 0.75-0.90, good reliability; 0.50-0.75, moderate reliability; and 0-0.5, poor reliability ^(7)^. Pearson’s correlation examined the associations between the Balancer and FSIQ and the four composite scores of the WISC-IV. The strength of Pearson’s correlation coefficients (r) was interpreted using the following guidelines: 0.9-1.0, very strong correlation; 0.7-0.9, strong correlation; 0.5-0.7, moderate correlation; 0.3-0.5, weak correlation; and 0.0-0.3, negligible correlation. The Friedman test evaluated significant differences across the three time points for each task, and *post hoc* analyses employed the Wilcoxon signed-rank test with Bonferroni-Holm adjustment to evaluate the differences between each time-point pairing ^(8)^. The Bland-Altman plots were used to compare the Balancer index scores with the relevant WISC-IV scores over three test administrations for each task to analyze the agreement between the Balancer and WISC-IV. The vertical axis shows the difference between the FSIQ and the index score (FSIQ − index score), whereas the horizontal axis presents the mean of the FSIQ and index scores (FSIQ + index score)/2. Analyses were conducted using the R software (version 3.6.1; R Foundation for Statistical Computing, Vienna, Austria). Statistical significance was set at p < 0.05.

## Results

### Reliability

The test-retest reliability results for both raw and age-adjusted index scores across the nine tasks are presented in Table 1. Good reliability across all three test sessions was demonstrated for both the raw and index scores of Catch the Mole and Speed Touch. Good reliability across selected pairs of test administrations (e.g., first to second and second to third) was evident for the raw scores of Drag Race, Matching Words, and Follow the Order. However, the reliability over all three test sessions was slightly lower. Moderate reliability across all three administrations was exhibited for the raw scores of Count the Blocks, Drag Race, Which Picture?, and Follow the Order tasks.

**Table 1.** Test-Retest Reliability for Raw Scores and Index Scores on the Kids Brain Balancer.

	Raw Score ICC (95% CI)	Index Score ICC (95% CI)	Duration (days) Mean (SD)
Count the Blocks (n = 19)
All*	0.54 (0.28-0.77)	0.56 (0.30-0.78)
(1,2)	0.74 (0.46-0.89)	0.74 (0.45-0.89)	3.37 (5.14)
(2,3)	0.38 (−0.07-0.70)	0.43 (−0.002-0.73)	2.21 (3.10)
(1,3)	0.49 (0.06-0.76)	0.49 (0.07-0.77)	5.58 (5.78)
Same Shape (n = 21)
All	0.21 (−0.04-0.51)	0.25 (−0.01-0.54)
(1,2)	0.34 (−0.09-0.66)	0.32 (−0.11-0.65)	1.80 (4.33)
(2,3)	−0.01 (−0.42-0.42)	0.02 (−0.40-0.43)	0.48 (1.37)
(1,3)	0.28 (−0.16-0.62)	0.37 (−0.05-0.68)	2.29 (4.47)
Drag Race (n = 23)
All	0.52 (0.28-0.74)	0.48 (0.23-0.71)
(1,2)	0.76 (0.52-0.89)	0.71 (0.43-0.86)	2.64 (4.71)
(2,3)	0.37 (−0.04-0.67)	0.28 (−0.14-0.61)	2.77 (4.03)
(1,3)	0.43 (0.04-0.71)	0.47 (0.08-0.73)	5.41 (5.72)
Which Picture? (n = 21)
All	0.58 (0.33-0.78)	0.43 (0.16-0.68)
(1,2)	0.71 (0.41-0.87)	0.47 (0.08-0.75)	1.00 (2.25)
(2,3)	0.49 (0.10-0.76)	0.32 (−0.11-0.65)	3.57 (3.92)
(1,3)	0.55 (0.17-0.79)	0.49 (0.09-0.75)	4.57 (3.68)
Matching Words (n = 15)
All	0.44 (0.13-0.73)	0.38 (0.06-0.69)
(1,2)	0.68 (0.29-0.88)	0.78 (0.47-0.92)	2.00 (2.76)
(2,3)	0.42 (−0.08-0.76)	0.23 (−0.29-0.65)	3.93 (4.31)
(1,3)	0.22 (−0.29-0.65)	0.05 (−0.44-0.53)	5.93 (3.77)
Catch the Mole (n = 19)
All	0.78 (0.61-0.90)	0.80 (0.64-0.91)
(1,2)	0.93 (0.83-0.97)	0.88 (0.71-0.95)	4.16 (6.02)
(2,3)	0.71 (0.40-0.88)	0.75 (0.47-0.90)	2.00 (3.36)
(1,3)	0.74 (0.45-0.89)	0.79 (0.54-0.91)	6.16 (6.38)
Speed Touch (n = 18)
All	0.77 (0.58-0.90)	0.76 (0.57-0.89)
(1,2)	0.90 (0.76-0.96)	0.86 (0.67-0.95)	1.38 (2.38)
(2,3)	0.70 (0.38-0.88)	0.71 (0.39-0.88)	2.67 (4.16)
(1,3)	0.69 (0.35-0.87)	0.71 (0.39-0.88)	4.05 (4.47)
Follow the Order (n = 19)
All	0.72 (0.51-0.87)	0.71 (0.50-0.87)
(1,2)	0.68 (0.34-0.86)	0.68 (0.35-0.86)	3.58 (4.53)
(2,3)	0.87 (0.71-0.95)	0.88 (0.71-0.95)	4.42 (4.10)
(1,3)	0.55 (0.15-0.80)	0.52 (0.11-0.78)	8.00 (5.48)
Flashing Lights (n = 22)
All	0.49 (0.23-0.71)	0.49 (0.23-0.72)
(1,2)	0.50 (0.12-0.76)	0.55 (0.19-0.78)	0.90 (1.88)
(2,3)	0.41 (0.01-0.70)	0.40 (−0.01-0.70)	2.14 (5.35)
(1,3)	0.57 (0.21-0.80)	0.53 (0.16-0.77)	3.05 (6.26)

ICC, intraclass correlation coefficient; CI, confidence interval; SD, standard deviation. *All three sessions: (1,2) the first and second sessions; (2,3) the second and third sessions; (1,3) the first and third sessions.

### Correlations with WISQ-IV

Moderate correlations were observed between the FSIQ scores on the WISC-IV and the Balancer index scores on several tasks over three repeated test administrations (Supplementary Table 1). Specifically, Count the Blocks, Speed Touch, Follow the Order, and Flashing Lights exhibited moderate correlations with the FSIQ across testing sessions. The Follow the Order task demonstrated particularly strong convergence with the PRI, whereas the Speed Touch, Follow the Order, and Flashing Lights tasks were aligned with the WMI.

### Score profiles over repeated testing

The ICCs between the FSIQ scores on the WISC-IV and Balance Index scores ranged from poor to good across the nine tasks over the three test sessions (Table 2). Although relatively stable, the ICCs were highest in the first session for five tasks (Count the Blocks, Same Shape, Catch the Mole, Speed Touch, and Follow the Order) and highest in the third session for three tasks (Drag Race, Matching Words, and Flashing Lights).

**Table 2.** Intraclass Correlation Coefficient (ICCs) between the Full-Scale Intelligence Quotient (FSIQ) on the Wechsler Intelligence Scale for Children, Fourth Edition (WISC-IV), and Index Scores on the Balancer App across Three Test Sessions.

	First time ICC (95% CI)	Second time ICC (95% CI)	Third time ICC (95% CI)
Count the Blocks	0.57 (0.17-0.80)	0.50 (0.10-0.86)	0.45 (0.02-0.75)
Same Shape	0.45 (0.02-0.75)	−0.08 (−0.37-0.30)	0.23 (−0.11-0.56)
Drag Race	0.23 (−0.10-0.56)	0.20 (−0.24-0.56)	0.29 (−0.14-0.62)
Which Picture?	0.29 (−0.14-0.62)	0.34 (−0.04-0.65)	0.23 (−0.19-0.59)
Matching Words	0.23 (−0.18-0.62)	0.25 (−0.28-0.67)	0.61 (0.16-0.85)
Catch the Mole	0.25 (−0.24-0.63)	0.13 (−0.35-0.55)	0.18 (−0.32-0.59)
Speed Touch	0.48 (0.04-0.77)	0.47 (0.05-0.76)	0.41 (0.01-0.72)
Follow the Order	0.55 (0.17-0.80)	0.49 (0.05-0.77)	0.46 (0.05-0.74)
Flashing Lights	0.40 (0.01-0.69)	0.56 (0.18-0.79)	0.58 (0.23-0.80)

ICC, intraclass correlation coefficient; CI, confidence interval; FSIQ, Full-Scale Intelligence Quotient; WISC-IV, Wechsler Intelligence Scale for Children, Fourth Edition.

**Figure 2.**
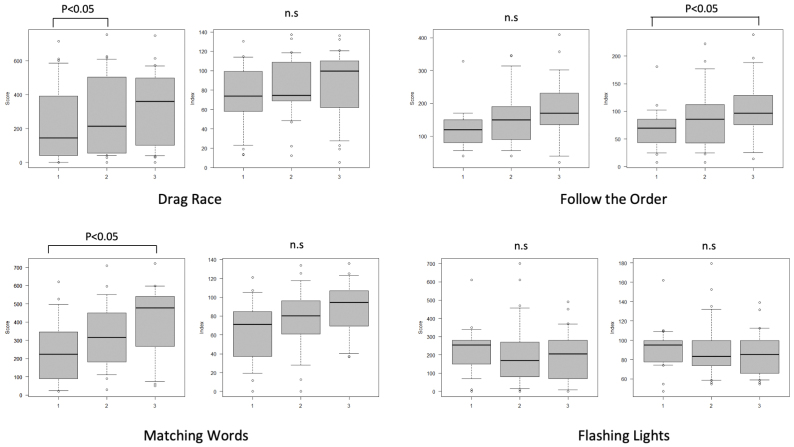
Score profiles over repeated testing.

The index score for Follow the Order and raw score for Matching Words significantly increased between the first and third test administrations (both p < 0.05) (Figure 2). The raw score for Drag Race was significantly higher in the second test than in the first. No other significant differences were observed between the testing occasions for any of the tasks.

### Score agreement between the Balancer and WISC-IV

**Figure 3.**
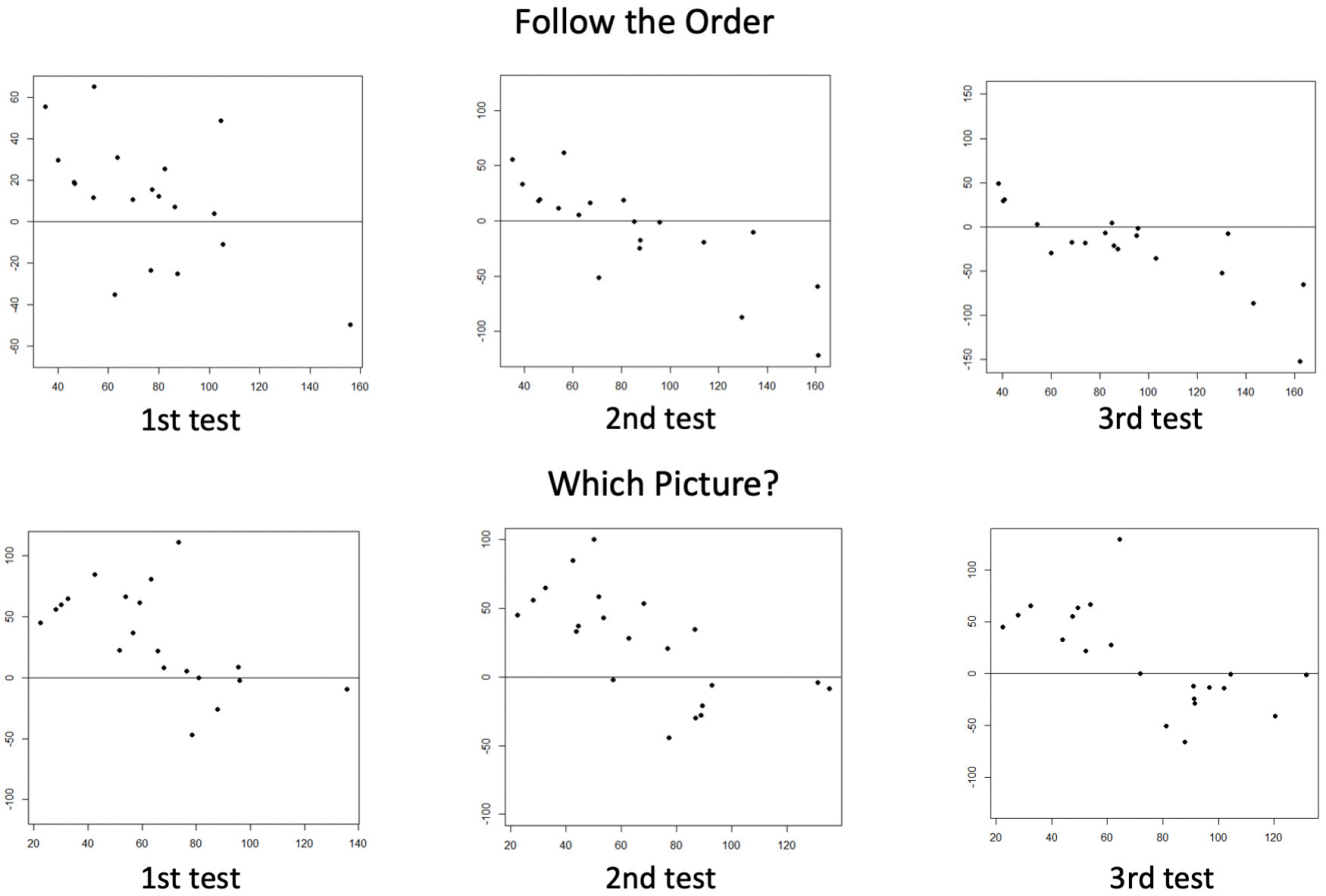
Score agreement between the index score of the Kids Brain Balancer (Balancer) and the Full-Scale Intelligence Quotient (FSIQ) obtained from the Wechsler Intelligence Scale for Children, Fourth Edition (WISC-IV), over repeated testing.

For Follow the Order and Which Picture?, the group with lower FSIQ or index scores showed mean differences approaching zero with successive testing (Figure 3). This indicates improved agreement between the Balancer and WISC-IV in children with lower baseline scores after repeated testing on these tasks. Proportional errors suggesting under- or overestimation by the Balancer were identified across both tests and score levels for all tasks, except for Same Shape, which exhibited a fixed negative bias.

## Discussion

This study provides preliminary evidence supporting the Balancer’s validity and test-retest reliability in assessing intellectual and cognitive functioning in children receiving special education services. Good-to-moderate reliability was observed across three repeated test administrations for raw scores and age-adjusted index scores in six of the nine tasks analyzed. Moreover, moderate-to-strong correlations were observed between the Balancer’s index scores and FSIQ as well as the composite index scores of the WISC-IV, enhancing the potential of the Balancer as a useful tool for intellectual assessment in children with developmental delays.

The Same Shape task exhibited relatively lower reliability than the other Balancer tasks, consistent with previous research on a comparable visual perception task among older adults ^(9)^. This indicates that fine figure-discrimination tasks, which require precise visual processing like the Same Shape task, may be inherently prone to performance variability over repeated tests.

Significant partial gains in performance on the Follow the Order, Matching Words, and Drag Race tasks indicate learning effects, whereby experience conducting tasks on successive occasions may enhance scores. Contrarily, most Balancer tasks exhibited score stability across the three test sessions, suggesting that repeated exposure is unlikely to threaten the validity of the Balancer in accurately representing children’s baseline intellectual levels or cognitive abilities in educational or clinical settings.

Score correlations between the FSIQ and Balancer across repeated testing exhibited mixed trends across specific tasks, with visuospatial and executive function tasks showing higher agreement with the FSIQ on initial testing. Working memory and language tasks showed improved convergence with the FSIQ in the later test sessions, suggesting that prolonged exposure better captures children’s cognitive capability. This indicates that for skills assessed more immediately, such as visuospatial skills, children’s functioning can be accurately represented after one trial. However, for capacities involving verbal and working memory storage, additional test familiarization may enable performance to more fully reflect potential.

The Bland-Altman plot analysis also revealed differing trends in children’s intellectual levels. The subgroup with lower assessed cognitive functioning (mean FSIQ and Balancer Index scores < 100) exhibited decreasing score differences over repeated testing in some tasks, suggesting that additional test familiarization improves score agreement. Contrarily, in the higher-scoring subgroup (mean FSIQ and Balancer Index scores > 100), the difference between FSIQ and the index score (FSIQ - index score) showed a consistent negative directional bias, which means the Balancer tended to overestimate the actual intellectual level lead by the FSIQ. Additional research is warranted to establish appropriate familiarization and correction protocols for Balancers based on a child’s baseline intellectual level.

The results indicated that the Balancer can be used as an assessment tool for cognitive/intellectual functioning among children receiving special education services. While some tasks exhibited learning effects impacting reliability and certain trends were observed based on the children’s initial intellectual levels, the detailed process data captured by the Balancer may prove valuable in supporting this diverse population. Notably, the Balancer’s advantage of enabling easy repeat testing allows the capture of individual differences in learning effectiveness across children and tasks. This granular tracking can help assess rehabilitation effectiveness and guide intervention planning, which is crucial in the formulation of the individualized support that is a mandatory aspect of special education. By easily creating materials that compare changes in cognitive function between arbitrary dates, these materials can be utilized to formulate and verify individualized support methods. Moreover, by sharing the data with all the parties involved (teachers, caregivers, psychologists, and other specialists) and parents, a shared understanding can be fostered to collaboratively support the child. Furthermore, this study did not exclude children with physical disabilities, although a detailed assessment was not yet conducted. The touchscreen interface of the Balancer opens up the possibility of cognitive assessment for individuals whose physical disabilities make existing paper-based intelligence tests challenging to administer. This touchscreen capability expands the applicability of the Balancer across the full spectrum of physical and cognitive functional diversities within special education settings.

This study has limitations that need to be acknowledged. The small sample has a gender imbalance and comes from a specialized population of children in special education without confirmed NDD diagnoses. This may limit broader applications, such as establishing reliability in larger general populations of children who meet the criteria for neurodevelopmental conditions and considering their individual developmental characteristics. In addition, general intelligence tests, including the WISC-IV, have been assessed in the general population, whereas the Balancer has only been examined in children receiving special education services. Flexibility in repeat-testing intervals may have influenced consistency and permitted task familiarization. Further investigation into how the duration between administrations impacts the measured reliability and potential learning benefits is warranted.

## Article Information

### Conflicts of Interest

Hashimoto K owns shares in LEDEX Corporation and receives royalties for the use of digital software copyrights. None of the other authors have any conflicts of interest to declare.

### Sources of Funding

This study was supported in part by the Japan Agency for Medical Research and Development (AMED) under Research Number 18gk0110013h0003. The funding source had no involvement in the design of the study; collection, analysis, and interpretation of data; writing of the manuscript; and decision to submit the article for publication.

### Acknowledgement

I would like to express my deep gratitude to my research advisors, Eisuke Inoue and Hokuto Moroboshi, for generously providing their expertise and thoughtful perspectives, which substantially enriched the interpretation and discussion of this study’s findings. I would also like to sincerely thank my collaborator, Takashi Watanabe, for the invaluable guidance and assistance with data processing.

### Author Contributions

Hashimoto K was responsible for the organization and coordination of the trial. Sugiyama T was the chief investigator and responsible for the data analysis. All authors contributed to the writing of the final manuscript.

### Approval by Institutional Review Board (IRB)

No. 2023-174-B in the Showa University Human Ethics Committee.

## Supplement

Supplementary Table 1 Correlations between the Full-Scale Intelligence Quotient (FSIQ) of the Wechsler Intelligence Scale for Children, Fourth Edition (WISC-IV), and the Kids Brain Balancer index score on several tasks over three repeated test administrations.
