# Supplementary material for: Information and Communication Technology-based Assessment for Children with Developmental Needs: Kids Brain Balancer
Source: JMA J. 2024 Aug 9;7(4):543–50. doi: 10.31662/jmaj.2024-0013 (PMC11543300; doi:10.31662/jmaj.2024-0013)
Supplement: Supplementary Table 1 — Correlations between the Full-Scale Intelligence Quotient (FSIQ) of the Wechsler Intelligence Scale for Children, Fourth Edition (WISC-IV), and the Kids Brain Balancer index score on several tasks over three repeated test administrations. [file 2433-3298-7-4-0543-s001.pdf]

Supplementary Table 1. Correlation coefficients between the Kids Brain Balancer index scores for various tasks and the Full-Scale Intelligence Quotient (FSIQ) as well as the composite index scores of the Wechsler Intelligence Scale for Children - Fourth Edition (WISC-IV), computed across three repeated test administration

		FSIQ			VCI			PRI			WMI			PSI		
		1	2	3	1	2	3	1	2	3	1	2	3	1	2	3
Count the Block	r	0.65	0.64	0.5	0.59	0.66	0.45	0.63	0.57	0.52	0.61	0.41	0.28	0.36	0.45	0.41
	p	<0.05	<0.05	<0.05	<0.05	<0.05	n.s	<0.05	<0.05	<0.05	<0.05	n.s	n.s	n.s	n.s	n.s
Same Shape	r	0.36	0.09	0.35	0.44	0.07	0.31	0.36	0.12	0.4	0.06	0.31	0.22	0.23	0.07	0.25
	p	n.s	n.s	n.s	<0.05	n.s	n.s	n.s	n.s	n.s	n.s	n.s	n.s	n.s	n.s	n.s
Drag Race	r	0.27	0.21	0.32	0.18	0.08	0.10	0.40	0.40	0.36	0.03	0.02	0.25	0.28	0.13	0.41
	p	n.s	n.s	n.s	n.s	n.s	n.s	n.s	n.s	n.s	n.s	n.s	n.s	n.s	n.s	n.s
Which Picture?	r	0.39	0.51	0.31	0.31	0.38	0.35	0.44	0.47	0.27	0.41	0.55	0.31	0.27	0.47	0.25
	p	n.s	<0.05	n.s	n.s	n.s	n.s	<0.05	<0.05	n.s	n.s	<0.05	n.s	n.s	<0.05	n.s
Matching Word	r	0.33	0.29	0.66	0.21	0.14	0.55	0.52	0.38	0.58	0.23	0.29	0.35	0.10	0.21	0.71
	p	n.s	n.s	<0.05	n.s	n.s	<0.05	<0.05	n.s	<0.05	n.s	n.s	n.s	n.s	n.s	<0.05
Catch the Mole	r	0.40	0.25	0.33	0.37	0.24	0.17	0.44	0.32	0.51	0.17	0.02	0.07	0.28	0.16	0.23
	p	n.s	n.s	n.s	n.s	n.s	n.s	n.s	n.s	<0.05	n.s	n.s	n.s	n.s	n.s	n.s
Speed Touch	r	0.62	0.64	0.56	0.40	0.36	0.28	0.52	0.52	0.52	0.68	0.72	0.59	0.65	0.73	0.63
	p	<0.05	<0.05	<0.05	n.s	n.s	n.s	<0.05	<0.05	<0.05	<0.05	<0.05	<0.05	<0.05	<0.05	<0.05
Follow the Order	r	0.64	0.69	0.72	0.45	0.52	0.47	0.73	0.75	0.80	0.49	0.49	0.51	0.69	0.65	0.73
	p	<0.05	<0.05	<0.05	n.s	<0.05	<0.05	<0.05	<0.05	<0.05	<0.05	<0.05	<0.05	<0.05	<0.05	<0.05
Flashing Lights	r	0.46	0.67	0.62	0.18	0.62	0.46	0.53	0.62	0.69	0.23	0.40	0.49	0.57	0.59	0.43
	p	<0.05	<0.05	<0.05	n.s	<0.05	<0.05	<0.05	<0.05	<0.05	n.s	n.s	<0.05	<0.05	<0.05	<0.05

Abbreviations: VCI, verbal comprehension index; PRI, perceptual reasoning index; WMI, working memory index; PSI, processing speed index; n.s, no significance.
